# Impact of mobile phone use on accidental falls risk in young adult pedestrians

**DOI:** 10.1016/j.heliyon.2023.e18366

**Published:** 2023-08-07

**Authors:** Paulo H.S. Pelicioni, Lloyd L.Y. Chan, Shuotong Shi, Kenny Wong, Lauren Kark, Yoshiro Okubo, Matthew A. Brodie

**Affiliations:** a School of Health Sciences, University of New South Wales, Kensington Campus, NSW 2052, Australia; b Neuroscience Research Australia, 139 Barker Street Randwick, NSW 2031, Australia; c School of Population Health, University of New South Wales, Sydney 2052, NSW, Australia; d Graduate School of Biomedical Engineering, University of New South Wales, Kensington Campus, NSW 2052, Australia

**Keywords:** Fall, Phone, Texting, Balance, Wearable, Road safety, Pedestrian, Dual-task, Gait, Accident, Walking

## Abstract

**Background:**

Mobile phone use is known to be a distraction to pedestrians, increasing their likelihood of crossing into oncoming traffic or colliding with other people. However, the effect of using a mobile phone to text while walking on gait stability and accidental falls in young adults remains inconclusive. This study uses a 70 cm low friction slip hazard and the threat of hazard to investigate the effects of texting while walking on gait stability, the ability to recover balance after a slip hazard and accidental falls.

**Methods:**

Fifty healthy young adults performed six walking tasks, and one seated texting task in random order. The walks were conducted over a 10-m walkway. Four progressive hazard levels were used: 1) Seated; 2) Normal Walk (walking across the walkway with no threat of a slip); 3) Threat (walking with the threat of a slip); and 4) Slip (walking with an actual 70 cm slip hazard). The three walking conditions were repeated twice with and without the mobile phone texting dual-task. Gait kinematics and trunk posture were recorded using wearable sensors attached to the head, trunk, pelvis and feet. Study outcomes were analyzed using repeated measures analysis of variance with significance set to *P≤.05*.

**Results:**

Mobile phone use significantly impaired postural balance recovery when slipping, as demonstrated by increased trunk sway. Mobile phone use negatively impacted gait stability as demonstrated by increased step time variability and decreased harmonic ratios. Increased hazard levels also led to reduced texting accuracy.

**Conclusions:**

Using a mobile phone to text while walking may compete with locomotor tasks, threat assessment and postural balance control mechanisms, which leads to an increased risk of accidental falls in young adults. Pedestrians should therefore be discouraged through new educational and technology-based initiatives (for example a “texting lock” on detection of walking) from texting while walking on roadside footpaths and other environments where substantial hazards to safety exist.

## 1 Introduction

The internet-of-things has resulted in people using their mobile devices to access emails, text and perform other tasks anywhere [[1], [2], [3], [4]], and it is common to see pedestrians using their mobile devices, such as mobile phones to text while walking. Consequently, the individual's attention may be divided between the locomotor and typing tasks [5,6]. The risks of texting while commuting include accidents caused by the distraction of typing [7,8].

Recent studies found that texting alters the walking performances of younger adults. This includes slower gait speeds and the head being held in a flexed position to view the device screen [[9], [10], [11], [12]] while walking. Texting may also reduce attention towards the external environment. For example, Nasar, Hecht and Wener [6] found that individuals occupied by their devices are more likely to cross the road into oncoming traffic. Using a virtual reality setup, Schwebel et al., 2012 [2] showed that texting diverted young adults’ attention from the street environment, increasing their likelihood of being hit by a car. People also presented larger lateral displacements while walking and texting than simple walking, which increased the possibility of colliding with other people and traffic [13].

Conversely, other studies reported that young adults could safely adapt their gait to incorporate mobile phone use and negotiate obstacles. For example, Hinton, Cheng and Paquette [14] observed that healthy young adults walking on a split-belt treadmill had their gait minimally affected and could maintain their texting performance. Similarly, Timmis et al., 2017 [15] reported that healthy young adults could successfully perform over-ground walking and texting concurrently with obstacle negotiation.

Considering these previous studies, the effect of mobile phone texting while walking on gait stability and accidental falls in young adults remains inconclusive. Slippery pathways often cause pedestrian falls, which may be exacerbated if the hazard level is unexpected or misjudged. Regarding balance recovery theory, both anticipatory gait adaptations (e.g., leaning forward prior to an expected perturbation) and reactive gait adaptations (e.g., time critical postural adjustments during a perturbation) contribute to successful avoidance of falling [[16], [17], [18], [19]] and may be trained [[20], [21], [22]]. Accordingly, preventing real-world falls requires the sufficient allocation of cognitive resources to threat assessment, anticipatory and reactive gait adaptations.

Developmental neuroscience further provides a theoretical frame work regarding why young adults are more likely to participate in risky behaviour [23,24] including texting while walking [[6], [7], [8]]. During adolescent brain development, the mismatch between the earlier development of the socioemotional network (sensitive to the stimuli and rewards of texting) and the later development of the cognitive control network (that regulates risky behaviour) [23] may result in young adults being less inclined to refrain from texting while walking and allocating sufficient resources to threat assessment. This oversight may be despite the presence of known hazards and education initiatives such as pedestrian signage, which aim to highlight the existence of such hazards.

In this paper, we therefore used wearable sensors to investigate the changes in postural balance recovery, gait stability and texting accuracy, caused by texting while walking with concurrent exposure to slip hazards. We aimed to investigate how the threat of slipping versus an actual slip hazard impacts both locomotor and texting performances. We hypothesised that the inclusion of four progressive hazard levels and uncertainty through the instructions “there may or may not be a slip” would require participants to allocate additional resources to threat assessment and enable better investigation of the differential impact of a texting dual-task on both anticipatory and reactive gait adaptations. Such knowledge is essential to delineate how using a mobile phone to text while walking may impact pedestrian accidents.

## 2 Materials and methods

### 2.1 Participants

In this cross-sectional study, we recruited 50 healthy young adults (22 females and 28 males with a mean age of 22.3 ± 1.8 years, an average height and weight of 169.5 ± 10.0 cm and 66.1 ± 12.8 Kg, respectively). Participants for this study were selected through convenience sampling of engineering students at the University of New South Wales. The eligibility criteria included having no musculoskeletal or neurological impairments that precluded exercise participation and no history of fracture in the past three months. The Human Research Ethics Committee at the University of New South Wales, Australia (HC17978) approved this study and informed consent were obtained prior to data collection in line with the Helsinki declaration.

### 2.2 Experimental setup

**Fig. 1:**
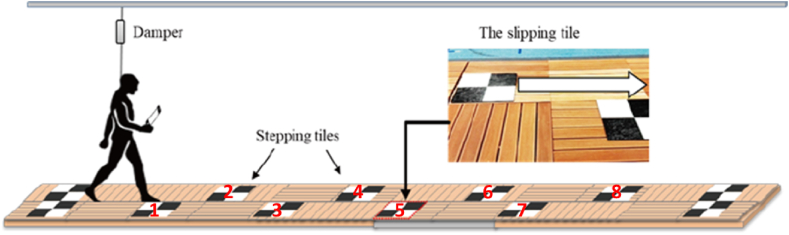
The perturbation walkway system with the 70 cm low friction slip hazard at tile five.

A 10-m perturbation walkway, consisting of 50 cm × 50 cm wooden decking tiles, was used [[20], [21], [22]]. Ten vinyl targets were laid on the walkway for participants to step on (Fig. 1). These step targets reduced the participants’ propensity to take smaller steps (as a form of anticipatory response) to an expected slip. The fifth vinyl target (third right step) was attached to a thin plate and low-friction bearings, sliding anteriorly up to 70 cm upon heel strike. Participants wore a full-body safety harness to ensure they did not fall to the ground (see Supplementary Video for set up).

Supplementary video related to this article can be found at https://doi.org/10.1016/j.heliyon.2023.e18366

The following is/are the supplementary data related to this article:

Experiment Re-enactment Experiment Re-enactment

**Fig. 2:**
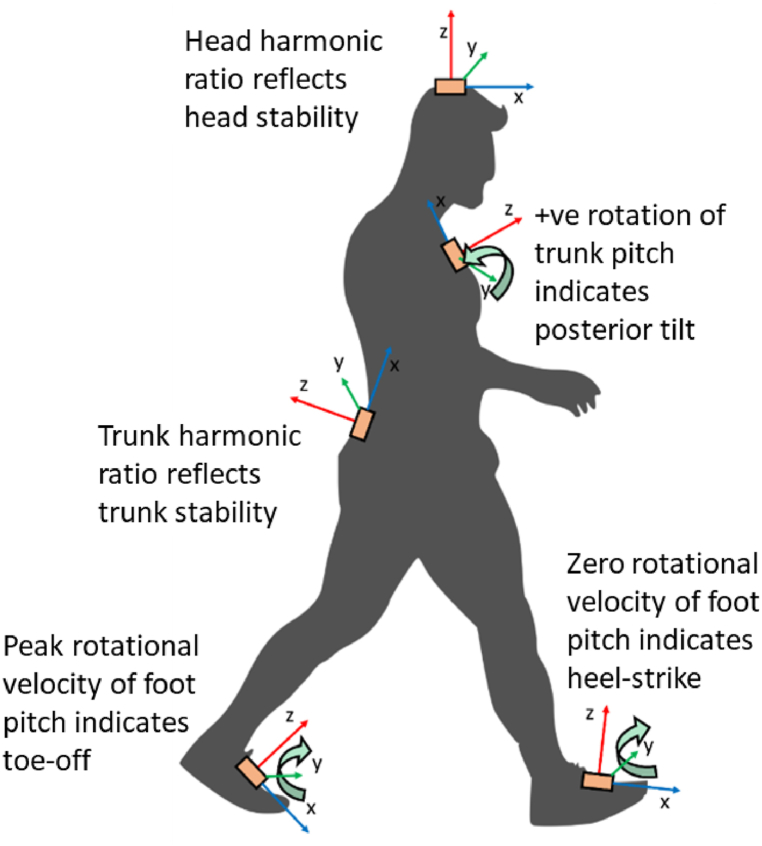
Sensor attachments, local sensor axes, trunk rotation convention and step detection.

Five Opal inertial measurement sensors (APDM, Portland, OR, USA) were attached to participants’ head (vertex), trunk (anterior to the xiphoid process of the sternum), waist (posterior to the sacrum) and dorsal aspect of the feet respectively (Fig. 2). Each Opal sensor housed a triaxial accelerometer (with a measuring range of ±10 g), a triaxial gyroscope (±2000 deg/s) and a triaxial magnetometer (±6 Gauss), with a sampling frequency of 128 Hz. We used these sensors to collect kinematic data.

### 2.3 Experimental procedures

Participants' gender, age, height and weight were recorded before the experimental tasks. We instructed participants to complete seven tasks in randomized order. The tasks included four progressive hazard levels: 1) Seated; 2) Normal Walk (walking across the walkway with no threat of a slip); 3) Threat (walking with the threat of a slip hazard that would not occur); and (4) Slip (walking with an actual 70 cm slip hazard tile that was unlocked). The three walking tasks were repeated twice with and without the texting dual-task. We instructed participants to prioritise walking and texting equally. The seated task was completed once to assess the undistracted texting performance.

For the Normal Walk condition (hazard level 2) we reassured participants that they would not encounter a slip hazard and locked the slipping tile. For the Threat condition (hazard level 3) we instructed participants that “there may or may not be a slip” and the slip tile was locked. For the Slip condition (hazard level 4) the same “there may or may not be a slip” instructions were given but the slip tile was unlocked.

For the texting dual-tasks, we instructed participants to use their smartphones to text the sentence “*The quick brown fox jumps over the lazy dog.*” exactly as written. We asked participants to turn off the predictive text function of their devices to enable an accurate count of correctly typed characters. We counted the total responses and correct responses by letter, including counting spaces, use of a capital T at the sentence start and a full stop (.) at the sentence end.

### 2.4 Data processing

**Fig. 3:**
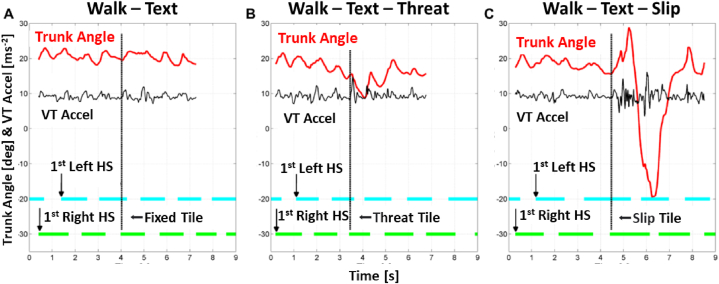
Sensor data shows an “accidental fall” from a slip hazard while texting. During each walk, postural balance was measured by trunk angle (in degrees), gait regularity was measured by vertical (VT) acceleration of the pelvis (meters per second squared) and heal strikes (HS) indicated at the start of each foot contact. Walk order was randomised. **[A]** Normal Walk condition (the participant was reassured that “there would be no slip”). **[B]** Threat condition (the participant was told “there may or may not be a slip” and they were blinded while we set the slip tile locking mechanism). For this walk the anticipatory gait adaptation was to progressively lean forward (downward trend of top line). At 3.4 s, the third right tile did not slip. **[C]** Slip condition (the participant was told “there may or may not be a slip”). The third right tile slipped. The participant lost balance posteriorly and then overcompensated anteriorly.

MATLAB (Natick, MA, USA) was used to process the wearable sensor data. We analyzed the eight intermediate steps, excluding the first (acceleration) and last (deceleration) steps (Fig. 3). We identified gait events through sagittal plane angular velocity peaks (toe-offs) and each foot sensor's subsequent zero crossings (heel strikes). We calculated: Step time (in seconds) as the duration between consecutive heel strikes; dual stance time as the percentage of walking time in which both feet were in contact with the ground; and step time variability as the standard deviation of step times (in milliseconds).

Data from the sensor located at the sternum were used to assess trunk orientation using sensor fusion techniques [25]. Next, we calculated the trunk pitch range by subtracting the minimum pitch angle (rotation about the mediolateral axis) from the maximum pitch angle during the walk. Increased range of trunk pitch was used to assess the risk of an accidental fall (if the participant had not been wearing a harness). Finally, we calculated the vertical harmonic ratio from the pelvis, trunk and head sensors as a combined measure of gait stability, symmetry and regularity [26,27].

### 2.5 Statistical analysis

To assess locomotor performance, we conducted a two-way repeated measures analysis of variance (rANOVA) for each gait measure. The effects on locomotor performance of the two dual-task scenarios (texting versus not texting) by the three walking hazard levels (Normal Walk; Threat; and Slip conditions) were investigated. The sitting task was not included in the locomotor analysis.

Regarding texting performance, we conducted a two-way rANOVA to examine the effects of the four hazard levels (Seated; Normal Walk; Threat; and Slip conditions) by the two measures of texting performance (total responses versus correct responses). Subsequently, we performed a one-way rANOVA to examine the effects of four hazard levels on the percentage of correct responses (number of correct responses divided by total responses).

If significant interactions or main effects were observed, post hoc paired t-tests were conducted. Differences in gait measures were also presented using Cohen's d. We used SPSS version 25.0 (IBM Inc., NY, USA) for statistical analysis. The level of significance for interaction and main effects was set to *P≤.05*. For post hoc analyses, Bonferroni corrections were used to account for multiple comparisons.

## 3 Results

### 3.1 The negative impact of mobile phone texting and hazards on falls risk

**Fig. 4:**
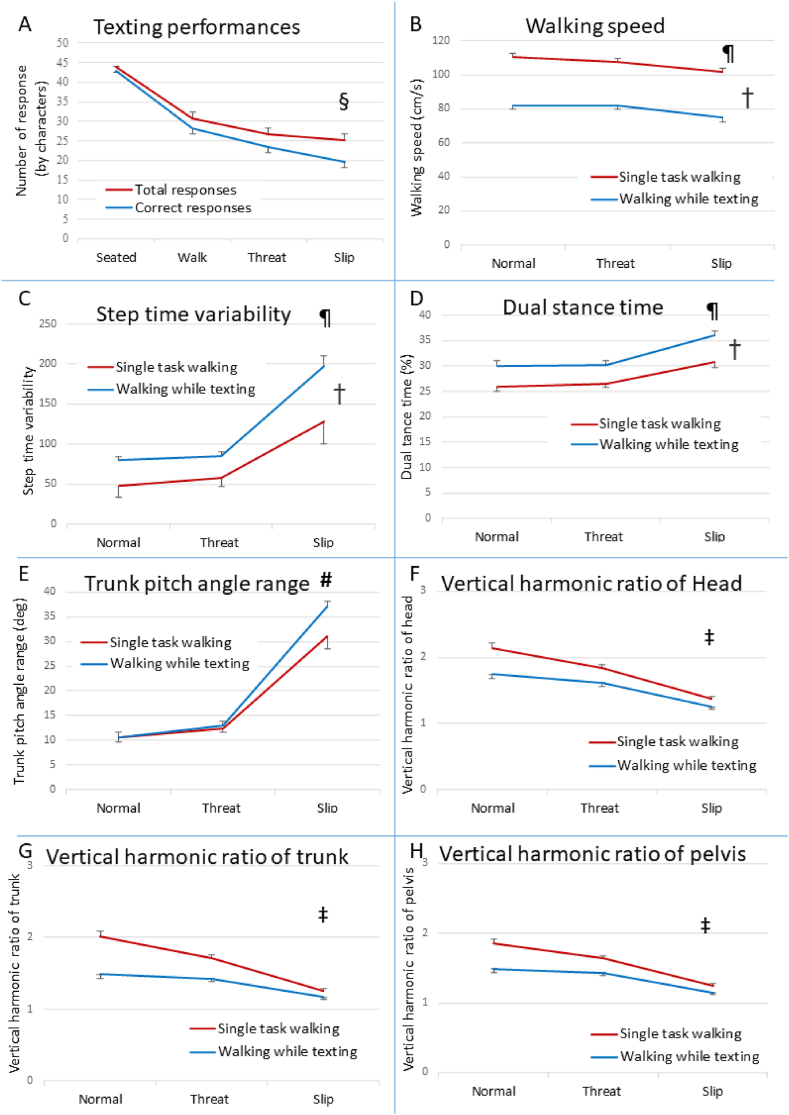
Changes in texting performance, gait and balance recovery (means ± 1 standard error). Texting while waking significantly impaired postural balance recovery and gait stability with increased hazard levels negatively impacting on texting accuracy. **[A]** (§) Indicates significant differences in texting performance between the Seated and Normal Walk; the Normal Walk and Threat, and the Threat and Slip hazard levels. **[B, C and D]** (¶) Indicates significantly (*P≤.05*) slower and more variable gait with longer dual stance times in the Slip condition compared to the Normal Walk and Threat conditions. (†) Indicates the texting dual-task led to significant (*P≤.05*) gait deterioration at all hazard levels. **[E]** (#) Indicates the texting dual-task led to significantly (*P≤.05*) greater risk of falling as indicated by increased trunk pitch range in the Slip condition, but not in the Normal Walk or Threat conditions. **[F, G and H]** (‡) Indicates a significant (*P≤.05*) interaction effect of reduced gait stability (indicated by lower harmonic ratio) between increasing hazard levels and the texting dual-task.

Texting while walking had a significant negative impact on all gait measures (Fig. 4 and Table 1). Increased hazard levels also significantly destabilised all gait measures. Post hoc analyses showed that the Slip condition produced the worst gait for all gait measures compared to the Normal Walk and Threat conditions (*P<.001*, Table 2). Comparing the Threat condition to the Normal Walk condition, an increased risk of accidental falls was indicated by an increased range of trunk pitch angle (*P<.001*) and decreased harmonic ratios of the head (*P<.001*), trunk (*P<.001*) and pelvis (*P<.01*).

**Table 1:** Main effects for hazard level and texting on gait measures. Data are mean (standard deviation).

Gait measures	Texting Task				Hazard level				
	Not texting	Texting	*F* (1,49)	*P-*value	Normal Walk	Threat	Slip	*F* (2,98)	*P-*value
Walking speed (ms^−1^)	1.06 (0.12)	0.80 (0.14)	247.3	**<.001**	95.2 (20.8)	94.5 (11.8)	88.6 (14.5)	20.7	**<.001**
Step time variability (ms)	80.8 (32.8)	128.9 (82.1)	21.2	**<.001**	71.4 (50.1)	75.2 (40.0)	167.9 (97.2)	50.5	**<.001**
Dual stance time (%)	27.8 (4.4)	32.0 (6.2)	36.0	**<.001**	28.0 (4.8)	28.3 (4.2)	33.4 (6.4)	62.0	**<.001**
Trunk pitch range (degree)	18.0 (5.5)	20.2 (7.1)	5.8	**.02**	10.6 (4.6)	12.7 (4.1)	34.0 (12.7)	163.0	**<.001**
Head harmonic ratio (vertical)	1.8 (0.2)	1.5 (0.3)	33.1	**<.001**	1.9 (0.4)	1.7 (0.3)	1.3 (0.2)	84.0	**<.001**
Trunk harmonic ratio (vertical)	1.7 (0.3)	1.4 (0.3)	69.9	**<.001**	1.7 (0.4)	1.6 (0.3)	1.2 (0.2)	71.6	**<.001**
Pelvis harmonic ratio (vertical)	1.6 (0.2)	1.4 (0.2)	39.9	**<.001**	1.7 (0.3)	1.5 (0.2)	1.2 (0.2)	65.7	**<.001**

Normal indicates the “normal walk” condition; Threat indicates the “walk with threat” condition; Slip indicates the “walk with slip” condition.

**Table 2:** Post-hoc paired t-tests for the main effects of hazard level and texting on gait measures. Results are expressed in Cohen's d with *P-*values in brackets.

	Walking Speed	Trunk Pitch Range	Dual Stance Time	Step Time Variability	Vertical HR of Head	Vertical HR of Trunk	Vertical HR of Pelvis
Normal Walk vs Threat	0.16 (.26)	−0.63 (<.001)	−0.16 (.26)	−0.09 (.51)	0.59 (<.001)	0.55 (<.001)	0.46 (<.01)
Normal Walk vs Slip	0.78 (<.001)	−1.97 (<.001)	−1.23 (<.001)	−1.07 (<.001)	1.69 (<.001)	1.48 (<.001)	1.47 (<.001)
Threat vs Slip	0.69 (<.001)	−1.71 (<.001)	−1.10 (<.001)	−1.05 (<.001)	1.39 (<.001)	1.33 (<.001)	1.21 (<.001)
Not texting vs Texting	2.22 (<.001)	−0.34 (.02)	−0.85 (<.001)	−0.65 (<.001)	0.82 (<.001)	1.18 (<.001)	0.89 (<.001)

HR indicates harmonic ratio; Normal indicates the “normal walk” condition; Threat indicates the “walk with threat” condition; Slip indicates the “walk with slip” condition.

The interaction effects between mobile phone texting and hazard levels were significant for trunk pitch angle range (*F(1.1,53.1)=4.23, P=.04*), head vertical harmonic ratio (*F(2,96)=6.6, P=.002*), trunk vertical harmonic ratio (*F(2,98)=15.4, P<.001*) and pelvis vertical harmonic ratio (*F(2,98)=7.136, P<.001*). Post hoc analysis (see Table 3) revealed that texting did not have a significant effect on trunk pitch angle in the Normal Walk (*P=.85*) and Threat conditions (*P=.41*) but had a significant effect in the Slip condition (*P=.03*). Texting had significant effects on the head, trunk, and pelvis vertical harmonic ratio in the Normal Walk (all *P<.001*), Threat (all *P<.001*), and Slip conditions (*P<.05*).

**Table 3:** Post-hoc paired t-tests for the interaction effects of hazard level and texting on trunk pitch angle, head, trunk and pelvis vertical harmonic ratios. Results are expressed in Cohen's d with *P-*values in brackets.

	Hazard level	Trunk Pitch Range	Vertical HR of Head	Vertical HR of Trunk	Vertical HR of Pelvis
Not Texting vs Texting	Normal Walk	−0.03 (.85)	0.77 (<.001)	1.03 (<.001)	0.74 (<.001)
	Threat	−0.12 (.41)	0.58 (<.001)	0.72 (<.001)	0.55 (<.001)
	Slip	−0.33 (.03)	0.33 (.02)	0.30 (.04)	0.40 (.01)

HR indicates harmonic ratio; Normal indicates the “normal walk” condition; Threat indicates the “walk with threat” condition; Slip indicates the “walk with slip” condition.

The wearable sensor data (Fig. 3) demonstrated how increasing hazard levels while texting may increase the risk of accidental falls in young adults. While experiencing a slip caused the most significant trunk destabilisation (Fig. 3C), anticipatory adjustments during the Threat condition was also observed to cause trunk destabilisation (Fig. 3B).

### 3.2 The negative impact of walking and hazards on texting accuracy

Texting accuracy was significantly impaired by increased hazard level (*F(3,147)=24.7, P<.001*, see Fig. 4 top left panel). Increasing the hazard level reduced the correct responses faster than the total responses, as indicated by a significant interaction between texting measures and hazard levels (*F(1.8,87.5)=14.2, P<.001*). One-way ANOVA on the percentage of correct responses was also significant (*F(1.9,92.9)=18.9, P<.001*). In the post hoc analyses, texting accuracy was the highest in the Seated condition (*P<.01*); texting accuracy during the Normal Walk condition was significantly better than during the Threat condition (*P=.04*); and texting accuracy was worst in the Slip condition (*P<.02*).

## 4 Discussion

Our findings demonstrate that walking while using a mobile phone to text, in the presence of an external hazard, increases the risk of accidental falls due to reduced gait stability and impaired ability to recover postural balance. Researchers and policymakers should therefore consider developing new planning, policies, educational and technology-based initiatives to reduce mobile phone texting in high-risk zones.

Our results agree with previous findings that texting while walking reduced walking speed and increased dual stance time [4,9,11]. Krasovsky, Weiss and Kyzoni [28] also observed that walking while texting led to lower texting accuracy. Our current study adds to this body of knowledge by demonstrating how texting while walking negatively impacts on gait stability and the ability to recovery postural balance after a slip. We further showed that there was a significant interaction between texting dual-tasks and hazard levels. Texting had the highest total responses and accuracy in the Seated condition, progressing with hazard level to the lowest accuracy in the Slip condition.

Our study provides new insights into how walking while using a mobile phone to text may compete for shared resources during concurrent exposure to hazards. Previous studies found that the dual tasks of walking and texting may divide attention [9,28], resulting in poor performances in both tasks [29] and that young adults may prioritise walking over texting [11,28]. It has been suggested that such prioritization depends on postural reserve, hazard assessment, skilfulness and task complexity, and individuals may be more likely to prioritise cognitive tasks under sufficiently safe circumstances [11].

During daily activities most accidents, including falls, contain an element of uncertainty, which may affect threat assessments. Exposure to slip hazards results in gait adaptations [16,19] that can be both anticipatory and reactive [[16], [17], [18]] and can be trained through repeated exposures with increasing uncertainly [[20], [21], [22]]. Anticipatory adaptations (such as leaning forward before a slip) may be most effective when the hazard is correctly predicted [18]. Reactive adaptations (including rapid postural adjustments and stepping) may be most effective during and after an unexpected or less predictable perturbation [[16], [17], [18]]. The random presentation order and the inclusion of the Threat condition (with no slip) in our study aimed to increase the ecological validity of the slip hazard by increasing the uncertainty. Therefore, our participants, in addition to dividing their attention between the locomotor and texting tasks, may have also had to consider the uncertainty of the slip hazard, had to make a threat assessment and had to decide how to allocate resources for both anticipatory and reactive gait adaptations and postural balance recovery.

In this study, we observed more cautious gait while texting as evidenced by reduced walking speed and longer dual stance time [Fig. 4]. These anticipatory gait adaptations may have indicated a combination of the participants’ perceived threat of texting while walking on their abilities to recovery balance and competition for shared resources. However, the anticipatory gait adaptations alone were insufficient to counteract the negative effects of texting on fall risks in young adults, as revealed by a significantly larger range of trunk angles following the slip events [Fig. 3, Fig. 4].

In daily life, uncontrolled variables including changing road conditions, uneven footpaths, crowds, bikes, cars and busses may significantly increase the negative consequences of texting using a mobile phone while walking. Conversely, in this study, participant safety was ensured by the experimental protocol, walking track and harness used. In daily life, the near instantaneous socioemotional rewards associated with smartphone use may make dual-task texting or video watching more addictive to young adults (and potentially young men) [[6], [7], [8]] who inherently may be more inclined to take risks [23,24]. It is therefore important to recognise the greater danger of mobile phone use in daily life compared to the laboratory environment and that educational initiatives including pedestrian signage may be less effective in young adults. One large-scale solution would be to use the “wearable” sensors in mobile phones to detect walking or driving. On detection of walking, for example [30,31], the mobile phone could activate a screen lock that temporarily prevents texting.

We acknowledge certain limitations. The sample population was limited to 50 healthy young adults with no known gait or motor impairments and the experiment was conducted under laboratory conditions with a safety harness. For safety reasons, the harness prevented actual falls from occurring and therefore precluded the collection of actual falls data and may have influenced behaviour. Through the consent process, participants were also informed of the study objectives and a combination of these factors may have affected the threat assessment and prioritization of the different tasks in each condition. The study was not powered for a three-way ANOVA and therefore gender differences regarding the different hazard levels and texting dual-task were not examined. Finally, to ensure the participant stepped on the fifth slip tile, step length was determined by the distance between tiles, which may have influenced the measurements of walking speed and step-time variability.

Future research should further investigate the impacts of mobile phone use in other cohorts including children, older adults and individuals with motor or cognitive impairments. Future research could investigate the risk of falls concurrently with real-life distractions outside of the laboratory setting. Future studies could further be powered to investigate gender differences. Large-scale technology-based solutions such as a “texting lock” on detection of walking could be incorporated into future interventional studies. Mobile phones have become ubiquitous and related accidents from improper use are likely to increase. More research regarding how age, gender, motor and cognitive impairments may impact mobile phone related accidents is therefore warranted.

## 5 Conclusions

Using a mobile phone to text while walking reduced the gait stability of young adults and increased their loss of postural balance when exposed to a slip hazard. Similarly, increasing the hazard level reduced texting accuracy, which together indicates some use of shared resources. Texting while walking may compete with locomotor tasks, threat assessment and postural balance control mechanisms, leading to an increased risk of falling. Pedestrians should therefore be discouraged through new educational and technology-based initiatives (for example a “texting lock” on detection of walking) from texting while walking on roadside footpaths and other environments where substantial hazards to safety exist.

## Author contribution statement

Paulo H S Pelicioni: Lloyd L Y Chan: Analyzed and interpreted the data; Wrote the paper.

Shuotong Shi: Kenny Wong: Performed the experiments; Analyzed and interpreted the data.

Lauren Kark: Analyzed and interpreted the data; Contributed reagents, materials, analysis tools or data.

Yoshiro Okubo: Conceived and designed the experiments; Analyzed and interpreted the data; Contributed reagents, materials, analysis tools or data.

Matthew Andrew Brodie: Conceived and designed the experiments; Performed the experiments; Analyzed and interpreted the data; Contributed reagents, materials, analysis tools or data; Wrote the paper.

## Data availability statement

Data will be made available on request.

## Declaration of competing interest

The authors declare that they have no known competing financial interests or personal relationships that could have appeared to influence the work reported in this paper.
